# Congenital Nephrogenic Diabetes Insipidus Presented With Bilateral Hydronephrosis and Urinary Infection: A Case Report

**DOI:** 10.1097/MD.0000000000003464

**Published:** 2016-06-03

**Authors:** Kewen Zheng, Yi Xie, Hanzhong Li

**Affiliations:** From the Department of Urology (KZ), the First Affiliated Hospital of Wenzhou Medical University, Wenzhou Medical University; and Department of Urology (KZ, YX, HL), Peking Union Medical College Hospital, Chinese Academy of Medical Science & Peking Union Medical College, P.R. China.

## Abstract

Nephrogenic diabetes insipidus (NDI) is a condition resulting from the kidney's impaired response to circulating antidiuretic hormone (ADH), leading to polydipsia and polyuria. Urinary tract dilatation caused by NDI is a rare situation. Here, we report a case of congenital NDI presented with bilateral hydronephrosis.

A 15-year-old boy complaining a history of intermittent fever was admitted to Peking Union Medical College Hospital. He voided 10 to 15 L of urine daily. Radiographic examination revealed severe dilatation of bilateral renal pelvis, ureter, and bladder. Urinalysis shows hyposthenuria.

He was diagnosed NDI since born. Transient insertion of a urethral catheter helped to relieve fever. Medical therapy of hydrochlorothiazide and amiloride was prescribed and effective.

Dilatation of urinary tract caused by diabetes insipidus is rare, but may be present in severe condition. Therefore, it is crucial for clinicians to perform early treatment to avoid impairment of renal function.

## INTRODUCTION

Diabetes insipidus (DI) is a condition characterized by the body's inability to conserve water or to concentrate the urine, leading to polydipsia and polyuria.^1^ Nephrogenic diabetes insipidus (NDI) results from the kidney's impaired response to circulating antidiuretic hormone (ADH).^1^ NDI causing severe urinary tract dilatation is a rare situation that is rarely published especially in children. This report presents a case of congenital NDI in a 15-year-old boy with severe urinary tract dilatation and urinary infection intermittently.

## CASE REPORT

A 15-year-old boy was admitted to Peking Union Medical College Hospital complaining of a history of intermittent fever with maximum body temperature of 41.0 °C for a year. Relevant past history included compulsive water drinking since infancy. He voided 10 to 15 L of urine daily and approximately 1 L once every 1 to 2 hours during day time. Physical examination was unremarkable except for fatness with a weight of 72 kg.

**FIGURE 1:**
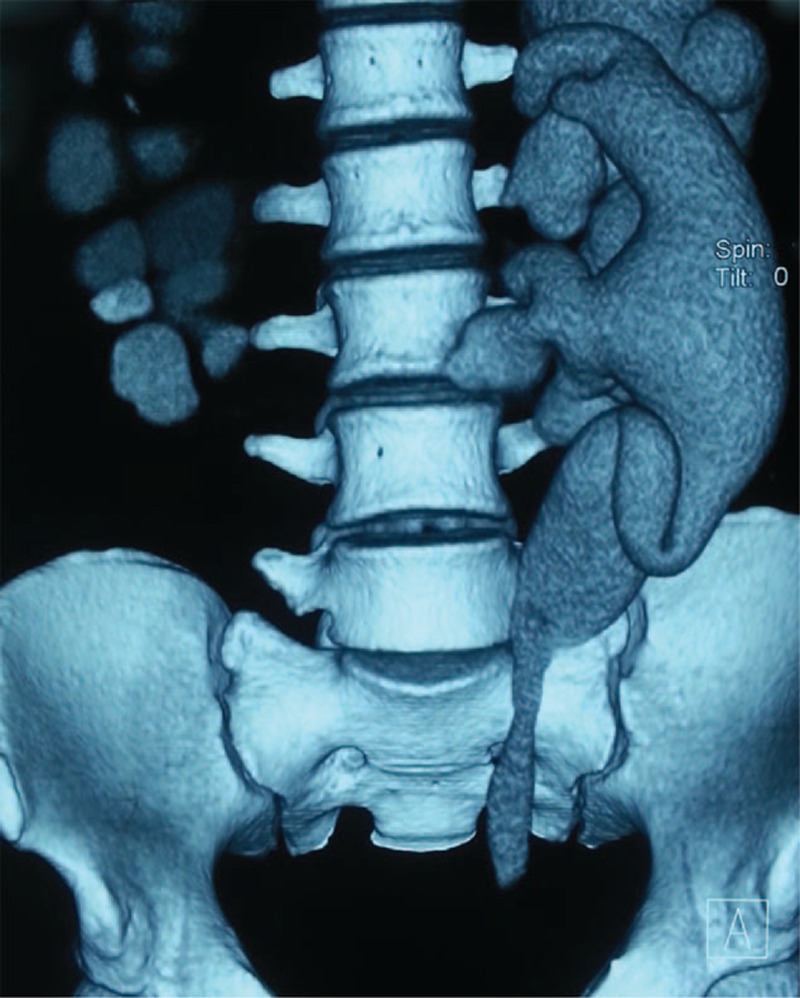
Three-dimensional computed tomography of the urography. Showing the marked bilateral dilatation of the ureter and renal pelvis.

**FIGURE 2:**
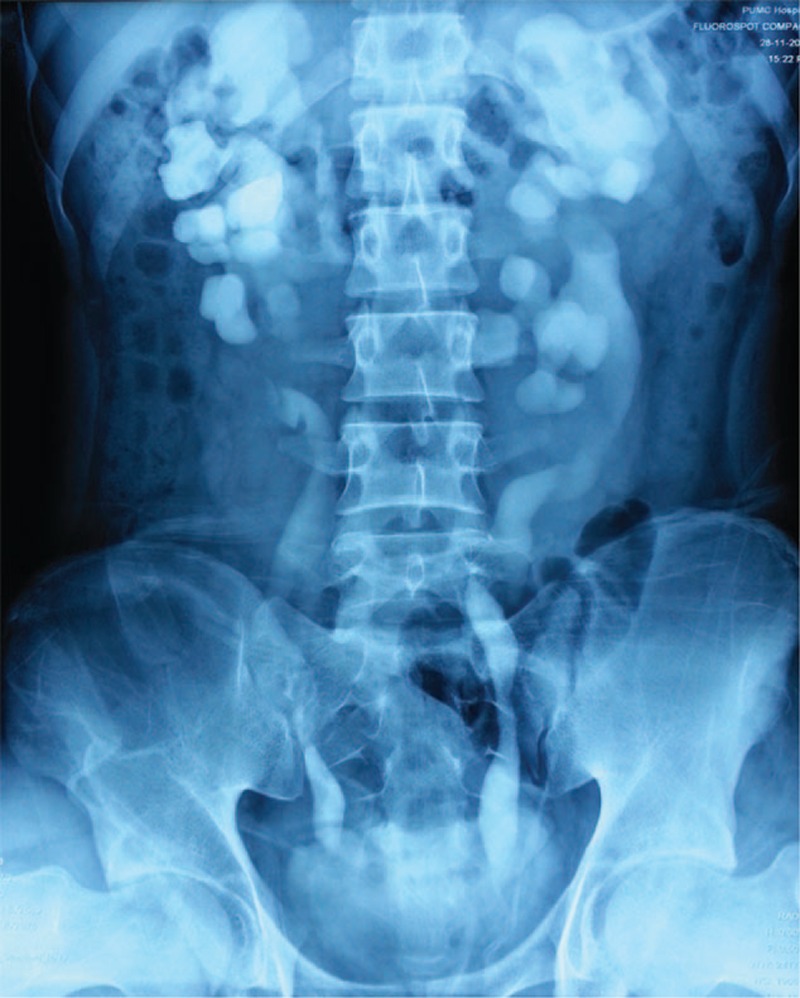
Retrograde pyelography. Showing the marked bilateral dilatation of the ureter and renal pelvis.

**FIGURE 3:**
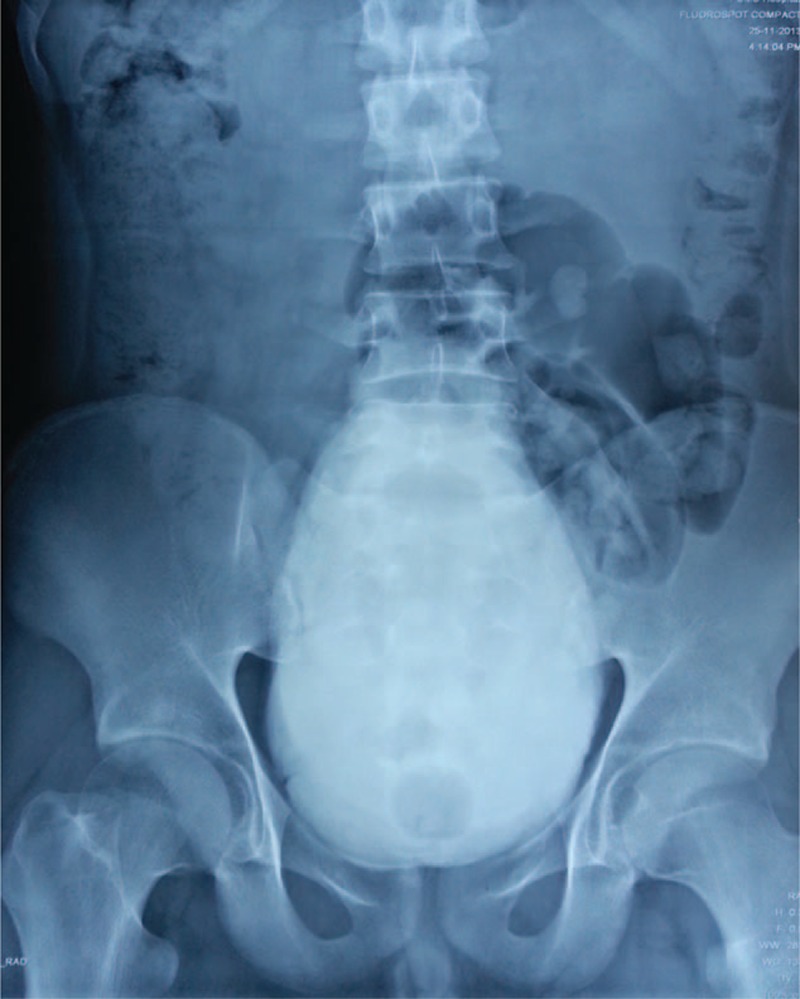
Cystoradiography. Bladder capacity exceeded 1000 mL. The bladder extended beyond the patient's umbilicus.

Urine osmolality was 148 mOsm/kg/day and urine specific gravity was lower than 1.005. Electrolytes including phosphorus, sodium, potassium, chloride, and calcium in 24-hours urine were lower than normal value, with 2.70 mmol/24 h, 52.78 mmol/24 h, 17.45 mmol/24 h, 40.95 mmol/24 h, and 1.01 mmol/24 h, respectively. Blood laboratory tests were normal except for mild chronic renal dysfunction (serum creatinine 1.14 mg/dL). Glomerular filtration rate was normal with 57.5 mL/min at right and 80.0 mL/min at left kidney. Radiology including CT urography (Figure 1), retrograde pyelography (Figure 2), and cystoradiography (Figure 3) showed non-obstructive, non-reflux dilatation of pelvis and ureter. Magnetic resonance imaging scan of the brain revealed no abnormalities in the sella turcica. Maximum flow rate (Qmax) was normal with 30.4 mL/s and postvoiding residual volume (PRV) was 500 mL.

**FIGURE 4:**
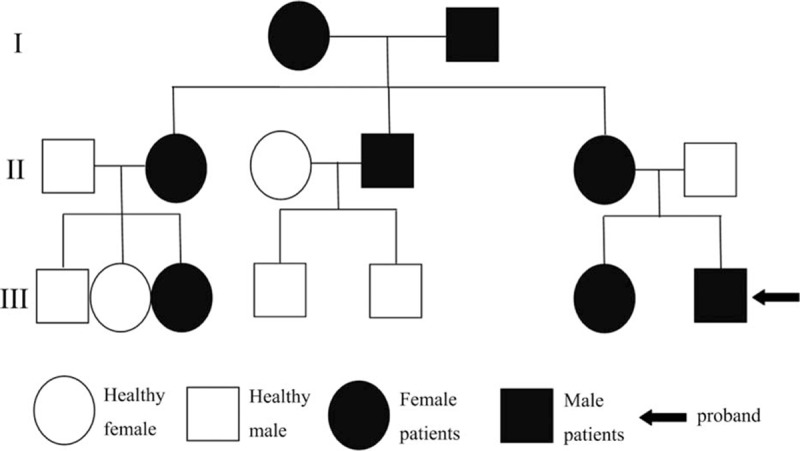
Pedigree chart of nephrogenic diabetes insipidus.

A water deprivation test was carried out and a diagnosis of NDI was made. Urine osmolality and a concomitant plasma osmolality tested on admission were 160 mOsm/kg and 308 mOsm/kg, respectively. A subsequent dose of 0.3 μg desamino-8-d-arginine vasopressin given intravenously failed to increase the urine osmolality. Family history was provided further to confirm the diagnosis of X-linked dominant NDI (Figure 4).

Fever was relieved after transient insertion of a urethral catheter. Medication therapy was carried out with a combination of hydrochlorothiazide (25 mg) and amiloride (2.5 mg) orally four times a day. Total urine volume was decreased by approximately 50% after the administration of drugs, reaching to 5000 to 8000 mL/24 h. In addition, salt restriction was recommended and the patient was taught to use abdominal pressure when voiding. During the 1-year follow-up with medication therapy, urine volume maintained between 5000 and 8000 mL/24 h. At the end of follow up, ultrasound examination showed that the width of left and right pelvis was 2.3 cm and 1.9 cm respectively, and serum creatinine remained 1.14 mg/dL.

The patient and his parents signed the informed consent and the ethics committee (composed by Guixin Qiu, Qi miao, Qiang Sun, and Zhigang Ji) approved the execution of the case.

## DISCUSSION

DI is classified into three types including NDI, central DI (CDI), and social or psychogenic DI. Water deprivation test helps to draw a diagnosis. About 90% of congenital NDI patients are caused by mutations in the V2 type of vasopressin receptor in the renal collecting duct, and are hereditary and X-linked.^1^ The other 10% of congenital NDI patients are inherited in autosomal recessive or autosomal dominant mode.^1^

DI resulting in urinary tract dilatation is considered to be rare. Dilatation may be present in severe DI.^3–7^ Several literature have been published to report about hydronephrosis, but mainly in the form of case reports and the etiology was not fully understood.^3–7^ The dilatation of urinary tract is an adaptive change as a result of the high flow, not obstruction which is confirmed by several reports.^2–5^ Qmax is reported to be normal in DI, which confirms its nonobstruction and differentiates it from neurogenic bladder. Pelvic pressure is increased in the presence of increasing diuresis, thus causing hydronephrosis, infection, and impairment of renal function in the untreated cases of severe condition. Sung and Lin^4^ reported that uncontrolled NDI may result in marked hydronephrosis, which may subsequently cause local renal hypoxia, increased erythropoietin production, and polycythemia. Besides hydronephrosis, the high voiding load leads to distention of bladder that is considered to impair bladder contractility.^2^ In fact, megacystis was reported in many published severe cases and also in our case.^7^ Furthermore, because of social embarrassment, patients’ voluntary urine retention tends to be a factor which aggravates bladder distention and pressure.

Treatment modalities such as diuretics, urethral catheterization, or cystostomy, and transurethral incision of the bladder neck are available to DI. Thiazide diuretics in combination with the potassium-sparing diuretic amiloride is helpful to diminish the degree of polyuria in patients with NDI. About 50% of total urine excretion deduction was observed with regular medication therapy.^6,7^ Besides, a high PRV was reported in most cases of DI, thus permanent or transient bladder drainage procedure (urethral catheterization or cystostomy) is necessary to prevent the situation from getting worse.^3^ Colliver et al^3^ reported four cases who underwent an insertion of a cystostomy button to improve bladder drainage and alleviate progressive renal decline with good resolution of incontinence and improvement in independence and quality of life. In addition, some authors conducted surgical procedures such as transurethral incision of the bladder neck to reduce the postvoiding residual volume (PVR) and relieve the symptoms of repeated urinary tract infection.^7^ However, it must be realized that surgical procedures are only adjuvants to medicinal therapy.^7^

In conclusion, we report here a case of congenital NDI with bilateral non-obstructive hydronephrosis that was diagnosed by water deprivation test and typical clinical history and imaging. Urinary volume can be reduced by medication but may not to normal volume. Transient insertion of a urethral catheter or surgical procedures could be considered in patients with large PVR after treatment with medication.
